# Electronic selection of viable *Legionella* cells by a video-based, quantifiable dielectrophoresis approach

**DOI:** 10.1007/s10544-025-00762-1

**Published:** 2025-07-30

**Authors:** Madeline Altmann, Anders Henriksson, Peter Neubauer, Mario Birkholz

**Affiliations:** 1 Chair of Bioprocess Engineering, Department of Biotechnology, Technische Universität Berlin, Berlin, Germany; 2 Joint Lab Bioelectronics, Department of Biotechnology, Technische Universität Berlin, Berlin, Germany

**Keywords:** Dielectrophoresis, Legionella, Single cell analysis, Live vs. dead analysis

## Abstract

The accurate selection of living from dead pathogenic cells is crucial as exemplified in the context of detecting *Legionella* bacteria, which can be present in various water facilities and pose a threat to public health by causing severe respiratory problems. Traditional methods for *Legionella* detection, such as cultivation, are time-consuming, taking several days to yield valid results. Additionally, widely used bioanalytical methods like PCR lack the ability to distinguish between living and dead cells, leading to the potential for false-positive results. While dielectrophoresis has been proposed as a promising method for separating living and dead cells, our study contrasts with existing literature, revealing that the separation process and parameter characterization are non-trivial. In response to this challenge, our work introduces a novel, systematic approach of automated video analysis capable of quantifying the dielectrophoretic response of cells. By assigning a response coefficient to the dielectrophoretic effect at different conditions, our method identifies a narrow window for successful cell selection of viable *Legionella* cells from the non-pathogenic species *L. parisiensis* utilizing a microfluidic flow cell with top–bottom electrodes. These findings serve as a crucial pre-step in *Legionella* sensing, demonstrating applicability in experiments focused on the most relevant pathogenic species, *L. pneumophila*. Moreover, our method can be transferred to other cell types for quantitative detection of the dielectrophoretic response and identify optimal separation parameters.

**Supplementary Information:**

The online version contains supplementary material available at 10.1007/s10544-025-00762-1.

## Introduction

In 2023, a report from the European Centre for Disease Prevention and Control reported a resurgence of Legionnaires’ disease, sparked by a recent increase in cases across Europe without a clear cause (European Disease Prevention & Control 2023). Legionnaires’ disease, an acute illness falling under the umbrella of Legionellosis, is one of several diseases caused by the Gram-negative bacterium *Legionella*, most notably *L. pneumophila*. With incidences rising worldwide, extensive surveillance and reporting procedures have been in place for years to monitor the spread and effectiveness of disease control.

*Legionella* bacteria are ubiquitous in water and soil, capable of reproducing in various hosts, including protozoa, alveolar macrophages, and epithelial cells. The primary mode of transmission is through the inhalation of contaminated aerosols, commonly occurring in environments such as cooling towers, air conditioning systems and whirlpools (World Health Organization (WHO) n.d.).

A conventional approach to detect contamination involves the filtration of the complex sample followed by incubation on agar plates to promote growth of any *Legionella* cells present in a sample. This method has various drawbacks including a lengthy incubation period of up to ten days, bias for specific *Legionella* species and challenges in handling complex samples. Alternative methods like quantitative polymerase chain reaction (qPCR) offer faster detection and the ability to easily differentiate between *Legionella* species. However, qPCR also can detect dead cells and may be inhibited by compounds found in complex environmental samples (Toplitsch et al. 2021), making qPCR unsuitable for tracking the efficiency of treatment methods.

Likewise, biosensors based on optical systems (Cooper et al. 2009), electrochemical immunoassays (Ezenarro et al. 2020) or impedance measurements (Lei and Leung 2012) allow for rapid and specific detection of *Legionella* but cannot distinguish between living and dead bacteria. Other methods, such as utilizing the 16S rRNA for the detection of *Legionella*, although being able to distinguish between living and dead cells, face the problem of sensitivity, where enrichment becomes a crucial issue due to the targeted detection limit required (Leskelä et al. 2005). The ability to distinguish living from dead cells becomes crucial when assessing the actual risk associated with *Legionella* contamination. Dead cells may be present in water samples, but their contribution to the risk of disease transmission is negligible as the pathogenicity of *Legionella* relies on its capability to replicate within human cells (Khweek and Amer 2010). Thus, the development of methods capable of specifically detecting living *Legionella* cells is essential for more accurate risk assessments, timely public health interventions, and monitoring the effectiveness of sterilization measures.

In this context, we explore the potential of dielectrophoresis (DEP) as a method for the targeted selection of viable *Legionella* cells as a preprocessing step for further bioanalytical applications. DEP is the manipulation of particles in an inhomogeneous electric field, resulting in a polarizable particle moving either towards (positive DEP) or against (negative DEP) the field gradient based on the relative polarizability of the particle compared to the medium. This interaction is directly influenced by the frequency of the electric field. The DEP force is dependent on the particle diameter as well as the electrical properties of both the particle and the medium (Pethig 2017; Tian et al. 2024; Emmerich et al. 2022; Abt et al. 2020). As early as 1966, the first microbiological study on DEP force in cell suspensions demonstrated the distinctiveness of living and dead cells, at that time by Pohl and Hawk for the case of yeast cells (Pohl and Hawk 1966). Subsequently, the approach was developed for various other cases (Kasarabada et al. 2024; Guo and Zhu 2015; Salomon et al. 2011; Lapizco-Encinas et al. 2004; Favakeh et al. 2025) as well as techniques for assessing cell viability using DEP (Zhang et al. 2020).

Different studies employing various electrode geometries and DEP applications have demonstrated the capability to effectively separate yeast (Matbaechi Ettehad and Wenger 2021) as well as bacteria (Lapizco-Encinas et al. 2004) according to their vitality. Furthermore, DEP can also be used to address other challenges of *Legionella* detection, as demonstrated by Fatoyinbo et al. (Fatoyinbo et al. 2014) with the removal of interfering soil particles from bacterial cells in complex environmental samples. The application of positive DEP seems especially promising since it has shown to improve the sensitivity of optical biosensor systems based on the targeted concentration of cells in close proximity to the sensor’s waveguide (Petrovszki et al. 2021; Henriksson et al. 2020, 2021). Hence, our approach involves the utilization of a microfluidic flow cell featuring an integrated top-bottom electrode configuration, an electrode geometry as investigated and applied by the DEP group at Humboldt University since 1993 (Schnelle et al. 1157), allowing cells to directly attach to the electrodes. Our focus centered on identifying parameters that facilitated the capture of exclusively living cells on the electrodes (Gascoyne et al. 1993), enabling the passage of heat shock inactivated cells through the microfluidic channel. In this study, we investigated two distinct Legionella species: the predominant species associated with human disease, *L. pneumophila* (Talapko et al. 2022), and the non-pathogenic, S1 strain *L. parisiensis* (Lo Presti et al. 1997; Stout et al. 2007). The successful selection of living from dead cells for *L. parisiensis* in both tap water and demineralized water was achieved through quantifying the DEP response with a video-based method.

## Materials and methods

In this study, the dielectrophoretic response of two distinct *Legionella* species, namely *L. pneumophila* and *L. parisiensis*, was investigated. The inactivated *L. pneumophila* samples were prepared analogously to the protocol described below by a partner laboratory with S2 status (BioSolutions Halle GmbH, Halle, Germany). The samples were transported at 4 °C in their respective medium. Due to the S1 status of the laboratory housing the microfluidic setup, the analysis of viable *L. pneumophila* samples was not feasible in this study.

### Preparation of viable and non-viable *Legionella parisiensis*

*Legionella parisiensis* cells (strain PF-209C-C2, DSM 19216) were cultivated on *Legionella* GVPC selective agar plates (Thermo Fisher Scientific, Germany). The agar plates were incubated at 37 °C for a period of 3 days and were afterwards stored at 4°C.

For sample preparation, visibly grown colonies were selected and subsequently diluted to an optical density (OD_600_) of 0.3 in either tap water (0.8 Sm^−1^) or demineralized water (0.1 mSm^−1^). The OD_600_ was measured using a photometer (Ultrospec 2100 pro, Amersham Biosciences Europe, Germany) at a wavelength of 600nm. To create non-viable cells, samples were heated to 80 °C, a temperature chosen to mimic real-life heat shock conditions encountered during water system disinfection. Various durations of heat shock ranging from 1 to 23 h were selected. To assess the efficacy of the heat shock treatment in inactivating Legionella parisiensis, GVPC selective agar plates were employed. Samples subjected to the heat shock treatment were inoculated onto the selective agar plates and showed no colony formation.

Additionally, to evaluate cell membrane integrity and assess cell viability, the bacterial suspension was stained with the nucleic acid dyes Propidium iodide (PI, Sigma Aldrich, Missouri, USA) and 4’,6-diamidino-2-phenylindole (DAPI, Carl Roth, Karlsruhe, Germany). Working solutions for both dyes were prepared according to the manufacturer’s specifications. The PI (λ_ex_ = 535 nm, λ_em_ = 617 nm) working solution was used at a concentration of 0.1vol%, selectively staining cells with compromised membranes. Additionally, 0.15vol% DAPI (λ_ex_ = 350 nm, λ_em_ = 470 nm) was employed, capable of passing through intact cell membranes and staining all cells.

Stained bacterial samples were examined under a fluorescence microscope (Eclipse Ti2-A, Nikon, Tokyo, Japan) to assess both vitality and stain quality. To ensure the specificity of the staining procedure, the potential bleed-through of DAPI fluorescence into the PI channel was evaluated. Stained samples were observed to confirm absence of bleed-through, ensuring the accurate discrimination of stained populations.

### Microfluidic system

**Fig. 1:**
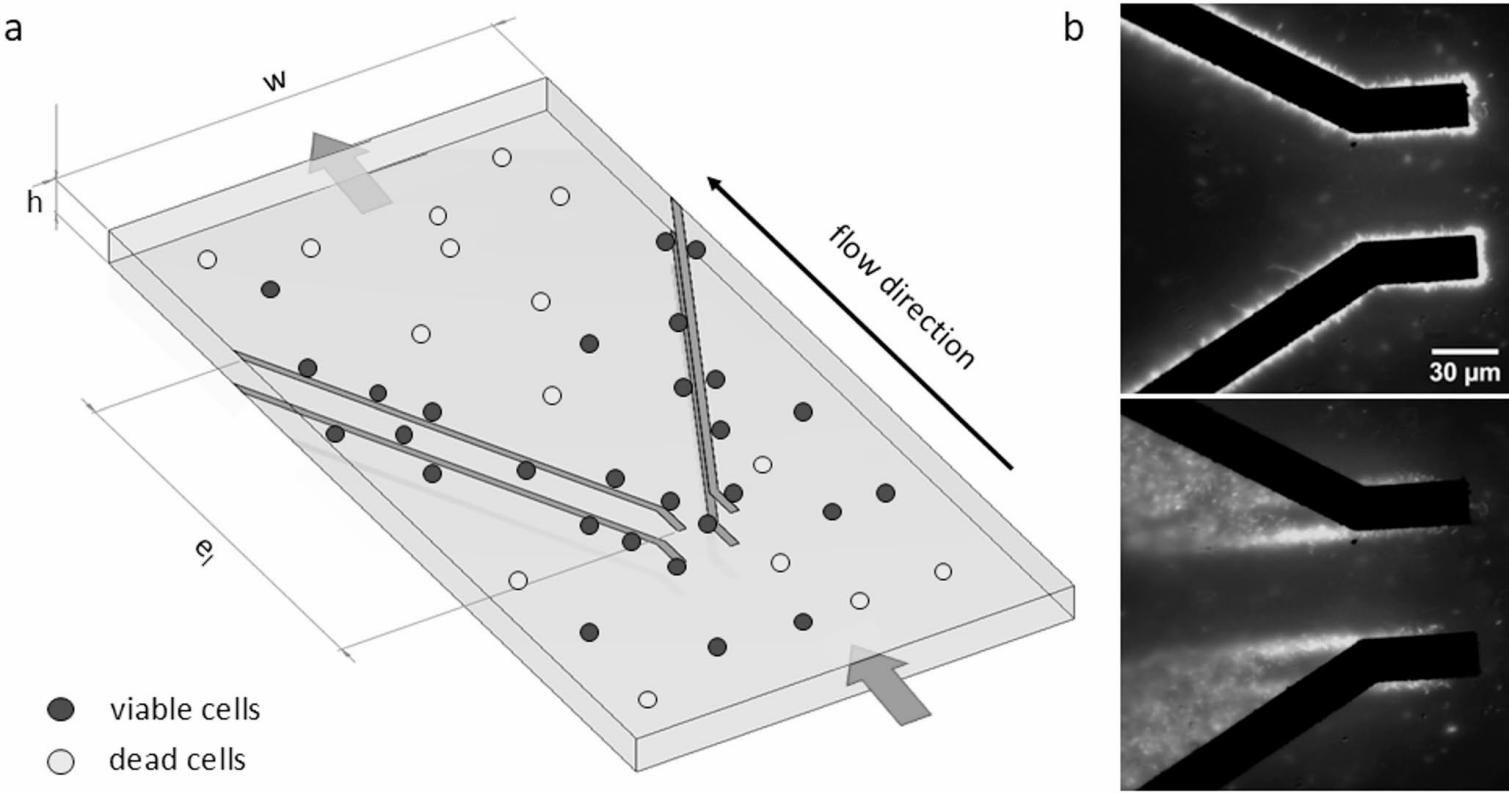
Microfluidic flow cell layout. (**a**) 3D model of the microfluidic device featuring two top–bottom electrode pairs 500 µm width (w), 50 µm height (h) and an electrode length of 550 µm (e_l_). Viable cells (blue) attach due to positive DEP to the electrodes, whereas non-viable cells (red) remain unattached and are passing without effect via the microfluidic flow. (**b**) Fluorescence microscopy image displaying the electrode array utilized on the microfluidic chip. The use of a Transmitted Light Microscope reveals only the bottom electrodes in the image. PI-stained cells (white) adhere to the electrodes during the active electrical field (top, here: 20Vpp, 2 MHz) and detach once the electrical field is switched off (bottom)

The microfluidic flow cell (GeSiM, Radeberg, Germany) used in this work was described prior in Birkholz et al. (2022). The flow cell is comprised of a 500 µm wide and 50 µm high microfluidic channel with various top-bottom Pt/Ti electrode arrays. In this study, an electrode geometry as depicted in Figure 1a was utilized to capture viable *Legionella* cells via positive dielectrophoresis (DEP), while non-viable cells continue to pass through the channel. To enhance the contrast between the microfluidic channel’s background and the diminutive Legionella cells, fluorescent dyes for cell staining were utilized (Figure 1b). For frequencies and voltages that induce a high DEP response, multiple layers of *Legionella* can adhere to the electrodes due to the strong dielectrophoretic force. After the electrical current is switched off, the cells detach from the electrodes and are carried away by the flux in the flow direction.

Example video: refer to [T1] 10.14279/depositonce-24071 in the supplementary

### Experimental setup

**Fig. 2:**
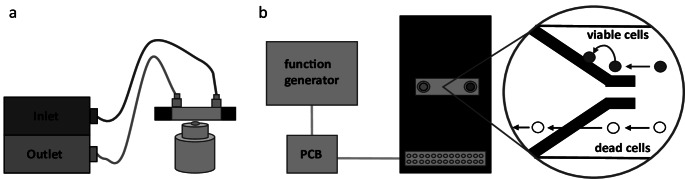
Schematic representation of the experimental setup for *Legionella* separation. (**a**) Cross-section of the microfluidic flow cell mounted on a transmitted light microscope. Connections to the inlet (blue) and outlet (orange) syringe. (**b**) Top view of the microfluidic flow cell with electrode connection pins, which can be individually addressed via the custom printed circuit board (PCB). Closeup reveals top–bottom electrode array with dead cells (orange) not showing a DEP response and passing through the channel, while viable cells (blue) attach to the electrode

A schematic representation of our experimental setup is shown in figure 2. The setup involved a microfluidic flow cell electrically connected to a function generator (AFG1062, Tektronix, Oregon, USA) using a custom-made printed circuit board (Boldt et al. 2021). The flow cell was further connected to two 2.5 ml microsyringes (SETonic, Ilmenau, Germany) for inlet and outlet flow. The flow cell was mounted on a transmitted light microscope (Eclipse Ti2-A, Nikon, Tokyo, Japan). The microscope operation and image acquisition were managed using the microscope’s own software, NIS Elements. For precise control of fluid flow, the two syringes were securely mounted on a microfluidics pump (Base 120, Cetoni, Korbußen, Germany). The pump’s operation, including flow rate adjustments and syringe control, was regulated using Qmix Elements.

Prior to operation and between samples, the fluidic channel was flushed with isopropanol for at least 15 min at a flow rate of 500 µl h^−1^, corresponding to an average flow velocity of 5.56 mm s^−1^. Afterwards, the channel was flushed with the respective sample at 500 µl h^−1^, before the flow rate was reduced to 10µlh^−1^. With the function generator, the observable frequency range extended from 0.5 to 25 MHz at 20 Vpp and reached up to 60 MHz at 10 Vpp or less. Frequencies below 0.5 MHz were not examined in this paper, because at frequencies below that, vortices were observed in the flow of the cells, which originated from the side walls of the microfluidic channel, and were attributed to electroosmotic effects (Ramos et al. 1999; Green et al. 2000). In general, the low-frequency range was not considered promising because hydrolysis may occur below 0.1 MHz causing the occurrence of gas bubbles, which must be avoided under all circumstances in microfluidic systems.

## Results & discussion

The successful selection of living *Legionella* from dead ones is a crucial goal for establishing a novel enrichment method for *Legionella* detection with the potential to be a viable alternative to time-consuming cultivation methods. Positive dielectrophoresis (DEP) is a non-invasive method that not only allows for the separation of bacterial cells based on vitality (Lapizco-Encinas et al. 2004), but also enhances the sensitivity of various biosensor types used in cell detection (Petrovszki et al. 2021), e.g. by bringing the cells in closer proximity to the detector.

In this study, we isolated untreated *Legionella* cells from inactivated ones through positive dielectrophoresis (DEP) by using a microfluidic flow cell featuring a top-bottom electrode configuration (as illustrated in fig. 1). Living and dead cells were stained with DAPI and then introduced into the channel, exposing them to voltages ranging from 5 to 20 Vpp at frequencies between 0.5 and 60 MHz. Initial experiments revealed a notable interaction of both living and dead *Legionella* cells with the electric field, manifesting an attachment of the cells to the electrodes and thus a positive DEP effect within the frequency range of 0.5-30 MHz at 10 Vpp in both demineralized and tap water. However, these observations did not identify specific parameters for effectively separating the cells. Recognizing the need for a more accurate and less subjective method, we propose a quantification approach for positive DEP.

### Quantification method for positive dielectrophoresis

To facilitate meaningful comparisons of the DEP effect across untreated *Legionella* and samples subjected to heat shock treatment, a robust and objective quantification method was imperative. PyImageJ, a Python wrapper for the image processing software ImageJ, was employed to achieve this, making use of videos taken of the fluidic channel during three consecutive phases: electrodes off-on-off.

**Fig. 3:**
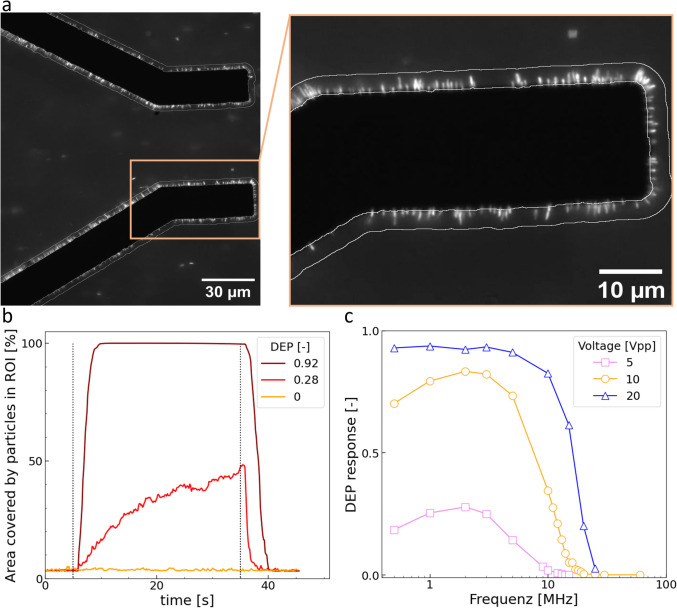
Quantifying positive dielectrophoresis using the DEP response coefficient. (a) Fluorescence microscope image of stained *Legionella* cells attached to bottom electrodes within the microfluidic flow cell (ROI outlined in white). (b) Example evaluation of particle area percentage in the ROI over 45 s, with the electric field activated at 5 s (first dotted line) and deactivated at 35 s (second dotted line). The DEP response coefficient is derived from the normalized integral of these curves, with baseline correction and slope criteria. Example curves: high DEP response (red), intermediate (orange), and none (yellow). (c) DEP response coefficient of *L. pneumophila* after 4 h heat shock in demineralized water within a 30 µm microfluidic flow cell, shown across different frequencies and voltages (n = 1)

A region of interest (ROI) was delineated around the electrodes, as illustrated in Figure 3a. Within this defined area (marked by a white border around the electrodes), the percentage of the ROI covered by fluorescent particles exceeding a predetermined threshold was quantified. For instance, a measurement may indicate that 50% of the ROI is occupied by particles surpassing the threshold (for additional details please consult the documentation available at [T2] 10.14279/depositonce-24072 in the supplementary).

In employing this methodology, 45-second videos were recorded to document fluorescence-stained cells within the microfluidic channel (for video examples, please refer to [T3] 10.14279/depositonce-24073). The initial 5 seconds served as a baseline, during which no electrical field was applied. Subsequently, the electric field was activated between top-bottom electrodes and maintained for 30 seconds, after which an additional 10-second period without electrical current was captured to observe cell detachment dynamics typical for positive DEP (Figure 3b): Very strong DEP effect, where multiple layers of Legionella attach to the ROI and saturation is observed (yellow curve), an intermediate DEP effect, where the %Area linearly increases (orange curve) and no DEP effect at all, where only background noise of cell debris or cells flowing through the ROI is recorded (red curve).

To describe the DEP effect for a particular condition, a DEP response is calculated by taking the integral of the %Area curve while the electrical current is turned off. The baseline is then subtracted by deducting the integral of the baseline scaled by a factor of 6 ((35-5)/5) to align with the same time length as the period when the electrodes are switched on. Finally, the DEP response coefficient is normalized by dividing by the maximal possible integral, in this case, 30s·100%Area, representing the entire duration and maximum coverage attainable.

The last 10 second period without electrical current serves as a control for establishing a cell detachment slope criterion. The rationale behind this criterion lies in the fact that if there is no detachment, indicating an absence of DEP, the %Area curve would not exhibit a monotonic decrease. Consequently, a lack of downward trend in the %Area curve during this control phase signifies a DEP integral coefficient of zero. Similarly, the algorithm assesses attachment within the ROI by examining the monotonic increase of the %Area curve during the activation of the electrical field.

The DEP response serves as a metric for visualizing the impact of DEP on cells under specific conditions. Illustrated in Figure 3c, for varying voltages, *L. pneumophila* exhibits a maximum DEP response at 2MHz following a 4-hour heat shock treatment. Notably, as the voltage decreases, the DEP effect diminishes. This is particularly evident at lower frequencies. At 20 Vpp, the inactivated cells exhibit positive DEP response across all relevant and adjustable frequencies for demineralized water achievable with the function generator.

Quantification methods of positive dielectrophoresis often rely on single image analysis (Lapizco-Encinas et al. 2004; Matbaechi Ettehad and Wenger 2021; Li 2002) or specific sensors capable of impedance measurements (Muhsin et al. 2022). In contrast, our method utilizes 45 s videos of fluorescence-stained cells, during which the electrical field is turned off and on, providing a comprehensive view of the dynamic DEP response.

Video analysis was chosen as the preferred method due to several considerations. While single-image quantification may be a robust method for analyzing the positive DEP effect on larger cells, as demonstrated by Ettehad et al. (Matbaechi Ettehad and Wenger 2021), we found that small cells like *Legionella* with dimensions of the order of 2-20 µm pose specific challenges. After a series of experiments, it became evident that the electrodes tended to accumulate cellular debris, impacting the clarity of individual images over time. Video analysis, on the other hand, allowed for a dynamic observation of the DEP effect, particularly as cells attach to the electrodes.

Furthermore, the strength of the DEP effect was more effectively discerned through video analysis. The speed at which the ROI reached 100% coverage with particles provided a quantitative measure of the DEP effectiveness. The method has its limitations, particularly in cases of very strong DEP responses leading to saturation in the region of interest (ROI) around the electrodes. Furthermore, as no negative DEP was observed in the examined frequency range, our method was not designed for cases where negative DEP might appear.

Nevertheless, we believe that this method provides a robust and straightforward approach for quantifying even weak DEP responses. It enables the identification of optimal parameters, such as those for cell selection, without requiring a specialized flow cell designed for sensing or additional measurement tools beyond a simple fluorescence microscope. The method is easily adaptable to various electrode geometries, organisms, and research goals.

### Selection of viable *Legionella parisiensis*

**Fig. 4:**
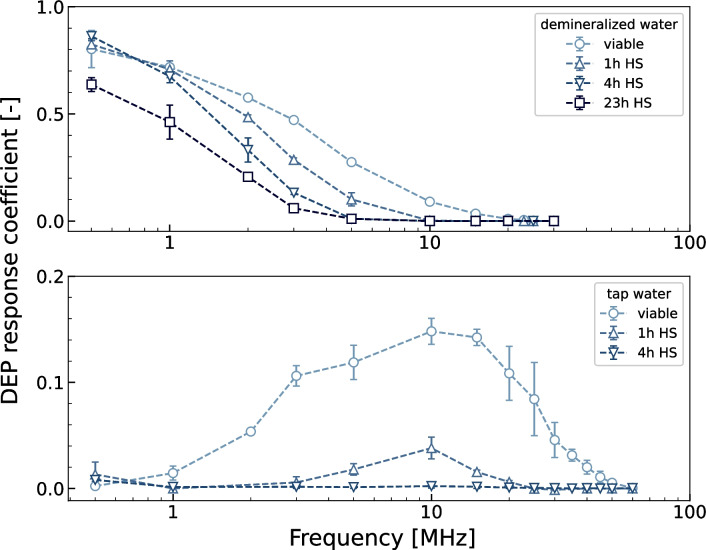
DEP response of *Legionella parisiensis* in demineralized and tap water. DAPI stained *L. parisiensis* in demineralized water (top) and tap water (bottom). DEP response coefficients at different frequencies at 10 Vpp. Heat shock (HS) treatment for 1 h, 4 h and 23 h respectively. Medium conductivity of 0.1mS m^−1^ and 0.8 S m.^−1^. n ≥ 3

As previously mentioned, at 20 Vpp in both tap water and demineralized water, all tested *Legionella* conditions strongly responded to positive DEP at every available frequency above 0.5 MHz (results not shown here). Through decreasing the voltage to 10 Vpp, selection of only viable cells was possible in both media (Figure 4). In demineralized water (0.1 mS m^−1^), all conditions exhibit a similar curve form reminiscent of a reverse sigmoid function. Even after 23 h heat shock treatment, the cells remain responsive to positive DEP. Notably, at 0.5 MHz the DEP response peaks and gradually diminishes with increasing frequencies. Extended heat shock treatments result in an overall lower DEP response coefficient, decreasing to zero for lower frequencies. At 10 MHz, cells subjected to heat shock treatments of 4 h or 23 h no longer display a positive DEP response. Although individual cells from the 1 h treatment still attach to the electrode, viable cells demonstrate a 52 times higher DEP response. Beyond 10 MHz, all heat shock treated samples exhibit zero DEP response, while viable cells maintain responsiveness up to 25 MHz.

**Fig. 5:**
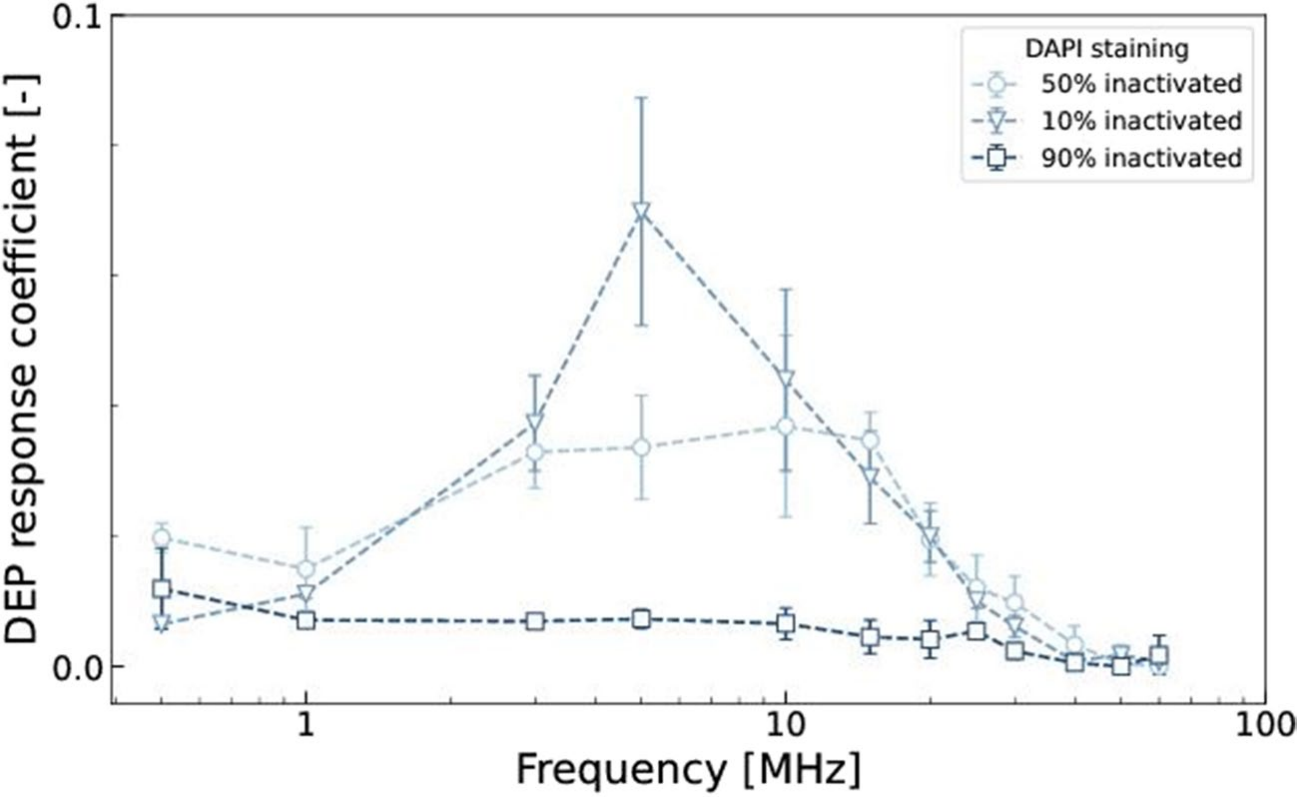
Selection of *Legionella parisiensis* cells from inactivated ones in tap water. *L. parisiensis* stained with DAPI (top) and PI (bottom) in tap water (0.8 S m^−1^). DEP response coefficients at different frequencies at 10 Vpp with n ≥ 3. Cell mixes of 10%, 50% and 90% inactivated cells

In tap water (0.8 S m^−1^), the DEP responsiveness of *Legionella* is overall lower than in demineralized water. However, viable cells show higher responsiveness to positive DEP than inactivated cells across the entire tested frequency range except at 0.5 MHz. The DEP coefficient versus frequency for viable cells and 1 h heat shock treated cells resembles a bell-shaped curve with a maximum at 10 MHz. After 4 h heat shock, only very few cells still respond to the electrode, with a maximum at 0.5 MHz. Viable cells exhibit responsiveness to DEP up to 50 MHz, demonstrating their ability to respond to the electric field across a broad frequency range, while only singular heat shock inactivated cells react above 20 MHz. Therefore, a selection of cells from inactivated *L.parisiensis* in tap water is recommended at 20-30 MHz at 10 Vpp.

As expected, the maximum generated DEP response was higher in demineralized water, since the difference between particle and medium conductivity is greater. Surprisingly, heat shock inactivated cells still respond strongly to positive DEP. As described by Lapizco-Encinas et al. (Lapizco-Encinas et al. 2004), the membrane of dead cells should have a significantly higher conductivity than the membrane of intact cells due to unobstructed ion exchange with the surrounding medium. Therefore, we anticipated that the response of dead cells would be greatly diminished, making the separation process more straightforward as described in literature. Therefore, we verified with inactivated *L. pneumophila* samples if our findings are species-specific. However, examinations of heat shock treated *L. pneumophila* showed very similar trajectories to inactivated *L. parisensis*, suggesting that this is not a *Legionella* species-dependent phenomenon (see in appendix fig. 6).

### Selection untreated *Legionella parisiensis* from mixed sample

Having established that untreated and inactivated *Legionella* exhibit distinct DEP responses, we proceeded to verify this phenomenon in mixed sample settings at various ratios of untreated to 4 h heat shock inactivated cells in tap water (Fig. 5). As anticipated, the overall DEP response closely resembled that of the pure samples, although the maxima in DEP response differs slightly in the 10% inactivated sample (compare Fig. 4). As expected, the DEP response decreases with higher proportions of inactivated cells in the mixture, confirming the earlier findings. Notably, this decrease was particularly pronounced across most frequencies, except at 0.5 MHz, where the response exhibited a unique behaviour consistent with previous observations.

The range between 20-25 MHz seems particularly interesting for DEP-supported biosensor applications. This is because when electrodes in the vicinity of a biosensor detection surface are operated with these frequencies, it is primarily the non-inactivated *Legionella* cells that would enter the biosensor’s detection area, while the inactivated cells would only be affected to a small extent. This means that only the actual pathogenic germs would be selected for detection and the sensor would not be flooded with unspecific signals. Such a concept is used, for example, in the photonic biosensor we have developed based on microring resonators (Henriksson et al. 2020), but also in other biosensor systems. This work has now successfully determined the frequency range to be used in the case of *Legionella*.

## Conclusion

In conclusion, our study underscores the capability of positive dielectrophoresis (DEP) to select even minute cells like *Legionella*. Furthermore, our findings reveal an unexpected resilience in the DEP response of inactivated *Legionella*. By employing a video-based quantification approach, a narrow window for distinguishing viable cells from their inactivated counterparts was found, showcasing the information richness compared to traditional image-based methods. While the specific testing focused on *L. parisiensis* due to the unavailability of viable *L. pneumophila*, the obtained results suggest the potential transferability of this methodology to other *Legionella* strains, including the more clinically relevant *L. pneumophila*.

The findings underscore the potential of DEP as a versatile technique that can be integrated into various facets of *Legionella* detection and analysis. Looking forward, DEP stands as a promising candidate for enhancing existing methods, such as serving as an enrichment strategy during sample preparation. In cell-based detection systems, DEP could be applied to attract living target cells from a crude sample to the detection surface, e.g. in combination with optical, electrical or acoustic sensors, or to repel the cells after the measurement for a repeated use of the device. As we continue to delve into innovative methodologies for pathogen detection, dielectrophoresis emerges as a powerful tool with the potential to shape the landscape of *Legionella* research and water safety analysis in the future.

## Supplementary Information

Below is the link to the electronic supplementary material.

Supplementary file1 (EPS 60 KB)

## Data Availability

Data is provided within the manuscript or supplementary information files T1-T3. T1 10.14279/depositonce-24071. T2 10.14279/depositonce-24072. T3 10.14279/depositonce-24073.
